# The impact of body mass index on adaptive immune cells in the human bone marrow

**DOI:** 10.1186/s12979-020-00186-w

**Published:** 2020-05-27

**Authors:** Luca Pangrazzi, Erin Naismith, Carina Miggitsch, Jose’ Antonio Carmona Arana, Michael Keller, Beatrix Grubeck-Loebenstein, Birgit Weinberger

**Affiliations:** 1 Department of Immunology, Institute for Biomedical Aging Research, University of Innsbruck, Rennweg, 10 Innsbruck, Austria; 2 Present Address: Center for Mind/Brain Sciences (CIMeC), University of Trento, Corso Bettini, 31 Rovereto, Italy; 3 Present Address: Private Kinderwunsch-Clinic Dr. J. Zech GmbH, Grabenweg 64, 6020 Innsbruck, Austria

**Keywords:** BMI, CMV, Human, Bone marrow, Peripheral blood, T cells

## Abstract

**Background:**

Obesity has been associated with chronic inflammation and oxidative stress. Both conditions play a determinant role in the pathogenesis of age-related diseases, such as immunosenescence. Adipose tissue can modulate the function of the immune system with the secretion of molecules influencing the phenotype of immune cells. The importance of the bone marrow (BM) in the maintenance of antigen-experienced adaptive immune cells has been documented in mice. Recently, some groups have investigated the survival of effector/memory T cells in the human BM. Despite this, whether high body mass index (BMI) may affect immune cells in the BM and the production of molecules supporting the maintenance of these cells it is unknown.

**Methods:**

Using flow cytometry, the frequency and the phenotype of immune cell populations were measured in paired BM and PB samples obtained from persons with different BMI. Furthermore, the expression of BM cytokines was assessed. The influence of cytomegalovirus (CMV) on T cell subsets was additionally considered, dividing the donors into the CMV^−^ and CMV^+^ groups.

**Results:**

Our study suggests that increased BMI may affect both the maintenance and the phenotype of adaptive immune cells in the BM. While the BM levels of IL-15 and IL-6, supporting the survival of highly differentiated T cells, and oxygen radicals increased in overweight persons, the production of IFNγ and TNF by CD8^+^ T cells was reduced. In addition, the frequency of B cells and CD4^+^ T cells positively correlated with BMI in the BM of CMV^−^ persons. Finally, the frequency of several T cell subsets, and the expression of senescence/exhaustion markers within these subpopulations, were affected by BMI. In particular, the levels of bona fide memory T cells may be reduced in overweight persons.

**Conclusion:**

Our work suggests that, in addition to aging and CMV, obesity may represent an additional risk factor for immunosenescence in adaptive immune cells. Metabolic interventions may help in improving the fitness of the immune system in the elderly.

## Introduction

Obesity is characterized by excessive accumulation of subcutaneous adipose tissue and visceral fat, which impairs overall health and promotes the development of several pathologies, including age-related diseases. Indeed, high body mass index (BMI) has been associated with cardiovascular diseases, type 2 diabetes, insulin resistance, and tumors [1, 2]. It has been shown that obesity is a cause of oxidative stress and chronic inflammation throughout the body, conditions which play a major role in the pathogenesis of diseases [3, 4]. Increased levels of pro-inflammatory cytokines such as IL-6 and TNF, and acute phase proteins have been documented in the peripheral blood (PB) of obese persons [5]. As adipose tissue and the immune system cooperate closely, either through the secretion of soluble mediators or after direct contact, accumulation of fat may influence the frequency and the phenotype of lymphocyte populations [6–8].

In the last years, several studies have demonstrated that the bone marrow (BM) plays an important role in the long-term maintenance of memory T cells and long-lived plasma cells [9–13]. In the elderly, the expression of pro-inflammatory molecules IFNγ and TNF and the levels of reactive oxygen species (ROS) are high in the BM [14]. In this situation, the expression of IL-15 and IL-6, which support the survival of highly differentiated CD8^+^ T cell subsets in the BM, is additionally increased. Furthermore, in the presence of latent cytomegalovirus (CMV) infection, IL-15 expression, as well as the frequency of highly differentiated CD28^−^CCR7^−^CD45RA^bright^ CD8^+^ T_EMRA_ cells in the BM, increase in comparisons to CMV seronegative persons [15].

Marrow adipose tissue (MAT) is located within the bone marrow microenvironment and is surrounded by hematopoietic and skeletal lineage cells. Subcutaneous white adipose tissue (WAT) is known to act as metabolic regulators, with the important functions of storing excess calories in the form of triglycerides and delivering fatty acids during fasting [16]. WAT additionally acts as endocrine organ, producing and secreting adipokines [17]. Recently, our lab described that MAT is characterized by elevated ROS levels and produces higher amounts of IL-15, IL-6 and TNF, in comparison to white adipose tissue (WAT) [18]. Furthermore, BM adipocytes were shown to impair the function of plasma cells [18]. Increased marrow adiposity has been described with obesity, at least in mice [19], and an influence of MAT on immune cells present within the BM can be expected. Nevertheless, it is unknown whether body weight may affect the frequency and phenotype of immune cells present within the marrow environment, and the production of molecules supporting the maintenance of adaptive immune cells in the BM. In the current study, the impact of body weight on BM niches supporting the survival of adaptive immune cells, as well as on the frequency of immune cell subsets in the BM, was assessed. In addition, the phenotype of T cell subsets was investigated in persons with different BMI, in both BM and PB. As the phenotype and the frequency of T cell subsets are strongly influenced by CMV, the comparisons were performed independently in CMV^−^ and CMV^+^ individuals. The expression of BM cytokines IL-15 and IL-6, known to support the survival of highly differentiated CD8^+^ T cells, and the levels of ROS, increased in overweight persons. The frequency of B cells, CD4^+^ T cells, as well as several T cell subsets were affected by BMI. In same cases, the changes in the BM were similar to the situation in the periphery. Thus, our work shows for the first time that body weight may affect both the maintenance and the phenotype of adaptive immune cells in the BM.

## Materials and methods

### Sample preparation

Samples were obtained from systemically healthy individuals who did not suffer from diseases known to affect the immune system. The donors comprised of 72 individuals (37 females, 35 males) aged between 31 and 89 years (mean age: 69.7 ± 12.9) and BMI between 20.2 and 43.5 (mean BMI: 28.9 ± 5.4). 35 samples were CMV seronegative and 37 seropositive. No age differences were observed between the two groups (age _CMV-_ = 69.8 ± 11; age _CMV+_ = 69.6 ± 13), and no correlations BMI/age were identified within the groups (r_s CMV-_ = − 0.15, *p* = 0.44; r_s CMV+_ = − 0.10, *p* = 0.51). The number of samples used in individual experiments is given in the figures and legends. For the isolation of bone marrow mononuclear cells (BMMCs), a fragment of *substantia spongiosa osseum,* which would otherwise be discarded, was collected during routine hip replacement surgery. The bone was further fragmented and treated with purified collagenase solution, constituted by the combination of a sulfhydryl protease (clostripain) and an aminopeptidase (CLSPA, Worthington Biochemical; 20 U/ml), in complete RPMI medium (RPMI 1640, Corning supplemented with 10% FCS, 100 U/ml penicillin, and 100 μg/ml streptomycin, Sigma) for 1 h at 37 °C. BMMCs were extracted using a filtered tube centrifugation step, and then purified using density gradient centrifugation (Lymphoprep®, Stemcell technologies). Paired samples of heparinised blood from the same donors were collected, and peripheral blood mononuclear cells (PBMCs) were purified by density gradient centrifugation.

### Cell culture and flow cytometric analysis

Immunofluorescence surface staining was performed by adding a panel of directly conjugated.

Abs to freshly prepared BMMCs and PBMCs. Dead cells were excluded from the analysis using a viability dye (Zombie AquaFixable viability dye or 7-AAD). After surface staining, cells were permeabilized using the Cytofix/Cytoperm kit (BD Pharmingen), and incubated with intracellular Abs. Cells were washed and measured using a FACSCanto II (BD Biosciences). Flow cytometry data were analysed using FlowJo v10 software.

To analyze IFNγ and TNF production, both BMMCs and PBMCs were stimulated for 4 h at 37 °C with 30 ng/ml PMA and 500 ng/ml ionomycin in the presence of 10 mg/ml brefeldin A (BFA; Sigma Aldrich). The production of IL-15 and IL-6 was assessed as previously described [14]. In summary, BMMCs were incubated for 12 h in the presence of 10 mg/ml brefeldin A. IL-15 and IL-6 mean fluorescence intensity (MFI) was measured with intracellular staining in the whole BMMC population.

The complete list of antibodies used for the experiments is shown in Suppl. Table 1.

### Measurement of ROS

BMMCs and PBMCs were incubated with the fluorescent dye dihydroethidium (Sigma-Aldrich) at a concentration of 1:250 in complete RPMI for 20 min at 37 °C. Cells were washed in PBS and measured with a FACSCanto II.

### Determination of CMV seropositivity

Antibodies against CMV were determined in the plasma of the donors included in the study using a commercially available ELISA Kit (Siemens).

### Statistical analysis

Spearman correlations were used to determine the statistical significance as indicated in the figure legends. Comparisons between groups were assessed with unpaired two-tailed t tests. Comparisons between PB and BM were performed with paired two tailed t tests. *p* values less than 0.05 were considered significant.

## Results

### Production of BM cytokines and reactive oxygen species (ROS) change with increased BMI

**Fig. 1:**
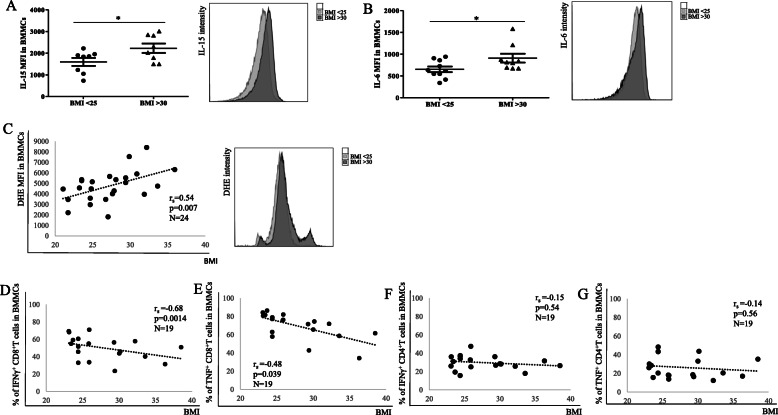
Levels of cytokines and ROS in the BM are affected by BMI. The mean fluorescence intensity (MFI) of (**a**) IL-15 and (**b**) IL-6 measured using flow cytometry in persons with BMI < 25 and BMI > 30 is shown. Unpaired t test, *p < 0.05. Data are reported as mean ± SEM. Relationship between ROS levels (=DHE MFI) (**c**), frequency of IFNγ^+^CD8^+^ T cells (**d**), TNF^+^CD8^+^ T cells (**e**) in BMMCs and BMI. Correlation between IFNγ^+^CD4^+^ T cells (**f**) and TNF^+^CD4^+^ T cells (**g**) in PBMCs and BMI. Spearman coefficient (r_s_), sample number (N), and *p*-values are reported in each graph. Representative histograms showing the intensity of IL-15, IL-6 and DHE in donors with BMI < 25 (BMI = 23.2) and BMI > 30 (BMI = 34.6) are shown

The BM microenvironment, which plays an important role in the maintenance of antigen-experienced adaptive immune cells, changes with age and CMV [14, 15]. To assess whether the BMI may also affect BM niches, the production of BM cytokines was measured in the BM of persons with different body weight (Fig. 1). The expression of both IL-15 and IL-6 in BMMCs was higher in the group BMI > 30, in comparison with lean persons (BMI < 25) (Fig. 1a-b). Levels of ROS and the pro-inflammatory molecules IFNγ and TNF increase in the BM with age [12]. When BMI was put in relationship with ROS levels in the BM, increased oxygen radicals were found in persons with higher body weight (Fig. 1c). Furthermore, reduced production of IFNγ and TNF by CD8^+^ T cells, but not by CD4^+^ T cells, was observed with higher BMI (Fig. 1d-g). No correlation was found between BMI and ROS levels, IFNγ^+^ and TNF^+^CD8^+^ T cells in PBMCs (Suppl. Fig. 1). Representative FACS plots showing IFNγ^+^ and TNF^+^CD8^+^ T cells in a lean and an obese donor in paired BM and PB samples are reported in Suppl. Fig. 2. Our results indicate that the expression of molecules supporting the maintenance of late differentiated adaptive immune cells in the BM and the production of T cell cytokines change in the BM with increased BMI.

### Immune cell populations are affected by BMI in the BM

**Fig. 2:**
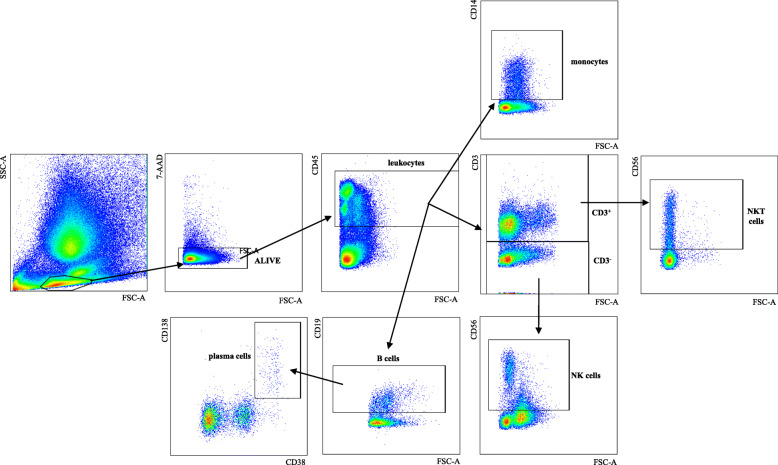
Gating strategy for the population of interest. Representative FACS plot with the gating strategy for monocytes, T cells, NKT cells, NK cells, B cells and plasma cells are shown

Associations between immune parameters and BMI have been described in the PB [8]. Whether immune cell populations in the BM change with increased body weight is unknown. As CMV is known to affect the phenotype of immune cells, we assessed the frequency of populations in BMMCs from CMV seronegative and seropositive persons in correlation with BMI (Table 1). The gating strategy for the populations included in the analysis is shown in Fig. 2. While natural killer (NK), natural killer T (NKT) cells, monocytes and plasma cells did not change, the overall B cell frequency in the BM increased with BMI in CMV^−^ persons (Table 1). No differences were observed for CMV^+^ donors, although B cell levels were similar in CMV^−^ and CMV^+^ persons (Suppl Fig. 3 A). While the levels of T cells and CD8^+^ T cells did not differ when overweight and lean persons were compared, the frequency of CD4^+^ T cells increased with BMI in CMV^−^ persons. Again, similar levels of CD4^+^ T cells were found in CMV^−^ and CMV^+^ persons (Suppl Fig. 3 B), but no significant correlations between BMI and CD4^+^ T cells were observed for CMV^+^ donors.

**Table 1:** Correlations of immune cell populations in in the human BM with BMI

	All		CMV^**−**^		CMV^**+**^	
	r_s_	*p* value	r_s_	*p* value	r_s_	*p* value
NK cells	0.05	0.72	0.16	0.46	0.08	0.69
NKT cells	0.07	0.64	0.13	0.55	0.03	0.89
monocytes	0.17	0.24	0.19	0.38	0.25	0.22
**B cells**	0.10	0.34	**0.41**	**0.01**	0.19	0.20
plasma cells	0.30	0.11	0.15	0.57	0.39	0.16
T cells	0.09	0.57	0.22	0.36	0.01	0.95
CD8^+^ T cells	0.07	0.58	0.03	0.88	0.02	0.92
**CD4**^**+**^**T cells**	**0.36**	**0.02**	**0.38**	**0.01**	0.12	0.59

Spearman correlation coefficients (r_s_) and *p* values for CMV^−^ and CMV^+^ persons and for the whole cohort are shown. *p* < 0.05 was considered significant. For NK, NKT, monocytes, T cells, CD8^+^ and CD4^+^ T cells N_CMV_^−^ = 30, N_CMV_^+^ = 35, N_all_ = 65, for B cells and plasma cells N_CMV_^−^ = 19, N_CMV_^+^ = 22, N_all_ = 41 (part of the same cohort). NK cells are defined as CD3^−^CD56^+^, T cells as CD3^+^, NKT cells as CD3^+^CD56^+^, monocytes as CD14^+^ and B cells as CD19^+^ leukocytes. Plasma cells are defined as CD38^hi^CD138^+^ B cells. Detailed gating strategy is shown in Fig. 2

Statistically significant values are shown in bold

In summary, although most of the immune cell populations in the BM were not affected, B cells and CD4^+^ T cells positively correlated with BMI, at least in CMV^−^ persons.

### CD4^+^ T cell subsets change with BMI

**Fig. 3:**
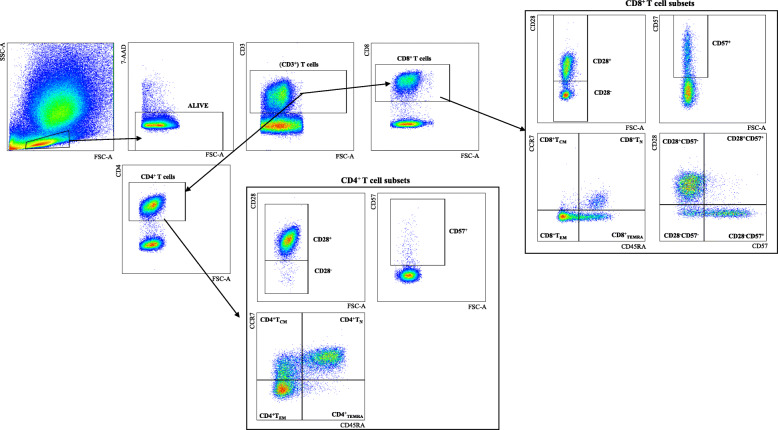
Gating strategy for the subsets of interest within CD4^+^ and CD8^+^ T cells. Representative FACS plot with the gating strategy for CD28^+/−^, CD57^+^, T_N_, T_CM_, T_EM_, T_EMRA_ within CD8^+^ and CD4^+^ T cells and the CD28^+^CD57^−^, CD28^+^CD57^+^, CD28^−^CD57^−^ and CD28^−^CD57^+^ subsets within CD8^+^ T cells are shown

We then aimed to investigate whether CD4^+^ T cell subpopulations may change with increased BMI, in both BMMCs and PBMCs (Table 2 and Suppl. Table 2). Gating strategy used to define the subsets of interest using flow cytometry is shown in Fig. 3. Using the markers CCR7 and CD45RA, we defined the four populations CCR7^+^CD45RA^+^ naïve (T_N_), CCR7^+^CD45RA^−^ (T_CM_), CCR7^−^CD45RA^−^ (T_EM_) and CCR7^−^CD45RA^+^ (T_EMRA_) within CD4^+^ T cells (Fig. 3). Interestingly, in the BM but not in the PB of CMV^+^ persons, CD4^+^ T_N_, CD4^+^ T_CM_ and CD4^+^ T_EMRA_ were positively associated, while CD4^+^ T_EM_ negatively correlated to BMI. In addition, in CMV^−^ donors, the frequency of CD4^+^ T_EMRA_ were higher in overweight persons. No differences were found for CD4^+^CD28^−^ and CD4^+^CD57^+^ T cells, neither in BMMCs nor in PBMCs. PD-1 is overexpressed in activated and/or exhausted T cells [20]. PD-1 expression in the whole BM CD4^+^ T cell population and in CD4^+^ T_EM_ cells negatively correlated with BMI, specifically in CMV^−^ persons. IL-7Rα expression on T cells is a marker for responsiveness to the T cell cytokine IL-7. Within CD4^+^ T cells, IL-7Rα was found to be increased with higher BMI. No differences were observed when the expression of PD-1 and IL-7Rα was assessed in the periphery (Suppl. Table 2).

**Table 2:** Correlations between CD4^+^ T cell subsets, expression of PD-1 and IL-7Rα within CD4^+^ T cell subsets in the BM and BMI

	All		CMV^**−**^		CMV^**+**^	
	r_s_	*p* value	r_s_	*p* value	r_s_	*p* value
**CD4**^**+**^**T cells (of CD3**^**+**^**)**	**0.36**	**0.02**	**0.38**	**0.01**	0.12	0.59
**CCR7**^**+**^**CD45RA**^**+**^**(CD4**^**+**^**T**_**N**_**)**	**0.45**	**0.002**	0.23	0.35	**0.51**	**0.02**
**CCR7**^**+**^**CD45RA**^**−**^**(CD4**^**+**^**T**_**CM**_**)**	**0.34**	**0.03**	− 0.18	0.46	**0.59**	**0.01**
**CCR7**^**−**^**CD45RA**^**−**^**(CD4**^**+**^**T**_**EM**_**)**	− 0.03	0.87	− 0.15	0.53	**− 0.58**	**0.01**
**CCR7**^**−**^**CD45RA**^**+**^**(CD4**^**+**^_**TEMRA**_**)**	**0.34**	**0.05**	**0.43**	**0.04**	**0.41**	**0.05**
CD4^+^CD28^−^	− 0.02	0.91	− 0.16	0.50	− 0.01	0.96
CD4^+^CD57^+^	0.00	1.00	0.16	0.42	0.27	0.91
**CD4**^**+**^**PD-1**^**+**^	−0.20	0.73	**− 0.41**	**0.04**	− 0.39	0.08
**CD4**^**+**^**IL-7Rα**^**+**^	**0.43**	**0.03**	0.37	0.12	0.03	0.91
**PD-1**^**+**^**CD4**^**+**^**T**_**CM**_	**−0.36**	**0.02**	− 0.21	0.39	− 0.22	0.34
**PD-1**^**+**^**CD4**^**+**^**T**_**EM**_	**−0.39**	**0.01**	**−0.49**	**0.03**	−0.16	0.48
CD57^+^CD4^+^T_EM_	−0.19	0.22	−0.02	0.93	0.14	0.55
PD-1^+^CD4^+^T_EMRA_	−0.33	0.10	−0.19	0.44	0.01	0.97
CD57^+^CD4^+^T_EMRA_	−0.03	0.85	−0.01	0.98	0.19	0.39

Spearman correlation coefficients (r_s_) and *p* values for CMV^−^ and CMV^+^ persons and for the whole cohort are shown. *p* < 0.05 was considered significant. For all subpopulations, N_CMV_^−^ = 20, N_CMV_^+^ = 19, N_all_ = 39. Detailed gating strategy used to define the populations are shown in Fig. 3

Statistically significant values are shown in bold

Taken together, our results indicate that, with increased BMI, the frequency and the phenotype of several CD4^+^ T cell subsets change in the BM, although no differences are present in the PB.

### CD8^+^ T cell subsets change with BMI

We next assessed whether CD8^+^ T cell subsets in the BM may additionally change with increased body weight (Table 3). Again, CD8^+^ T_N_, T_CM_, T_EM_ and T_EMRA_ subpopulations were gated using the markers CCR7 and CD45RA. While the frequency of CD8^+^ T_CM_ cells in the BM negatively correlated with BMI in CMV^−^ persons, CD8^+^ T_EM_ cells decreased with increased body weight only in CMV^+^ donors (Table 3). No significant differences were observed for CD8^+^ T_N_ and CD8^+^ T_EMRA_ cells. When the levels of the four subsets were measured in paired PBMC samples, CD8^+^ T_EM_ were highly reduced in the CMV^−^ group with increased BMI, but CD8^+^ T_CM_ cells, as well as CD8^+^ T_EMRA_ cells did not change in overweight compared to lean persons (Suppl. Table 3). A positive correlation between frequency of CD8^+^ T_N_ cells and BMI was also observed in the PB. With the markers CD28 and CD57, the populations CD28^−^, CD57^+^, as well as CD28^+^CD57^−^, CD28^+^CD57^+^, CD28^−^CD57^−^ and CD28^−^CD57^+^ were gated within CD8^+^ T cells in BMMCs (Fig. 2). Only in CMV^−^ persons, the CD28^+^CD57^−^ and CD28^+^CD57^+^ subsets negatively correlated, while CD28^−^CD57^−^ cells were positively associated with BMI (Table 3). No significant differences were found for the CD28^−^CD57^+^ subset. Similar results were found in PBMCs for CD28^+^CD57^+^ and CD28^−^CD57^−^ cells, while no changes were observed for the other two subsets (Suppl. Table 3). Taken together, our data show that several CD8^+^ T cell populations change in the BM with increased BMI, particularly in the absence of CMV. Overall, the frequency of memory CD8^+^ T cells may be reduced in overweight compared to lean persons, while highly differentiated CD28^−^CD57^−^ CD8^+^ T cells increase.

**Table 3:** Correlations of CD8^+^ T cell subsets in in the human BM with BMI

	All		CMV^**−**^		CMV^**+**^	
	r_s_	*p* value	rs	*p* value	rs	*p* value
CD8^+^ T cells	−0.07	0.58	−0.03	0.88	−0.02	0.92
CCR7^+^ CD45RA^+^ (CD8^+^T_N_)	0.25	0.10	0.00	0.72	0.34	0.10
**CCR7**^**+**^**CD45RA**^**−**^**(CD8**^**+**^**T**_**CM**_**)**	−0.03	0.78	**−0.42**	**0.05**	−0.03	0.88
**CCR7**^**−**^**CD45RA**^**−**^**(CD8**^**+**^**T**_**EM**_**)**	**−0.34**	**0.02**	−0.24	0.27	**−0.41**	**0.05**
CCR7^−^CD45RA^+^ (CD8^+^_TEMRA_)	0.08	0.61	0.23	0.30	0.01	0.94
**CD8**^**+**^**CD57**^**+**^	**−0.32**	**0.01**	**−0.33**	**0.05**	−0.27	0.19
CD8^+^CD28^−^	−0.18	0.23	−0.06	0.78	−0.11	0.62
**CD8**^**+**^**CD28**^**+**^**CD57**^**−**^	−0.21	0.13	**−0.34**	**0.04**	−0.16	0.44
**CD8**^**+**^**CD28**^**+**^**CD57**^**+**^	−0.19	0.16	**−0.41**	**0.02**	0.09	0.67
**CD8**^**+**^**CD28**^**−**^**CD57**^**−**^	**0.36**	**0.01**	**0.44**	**0.01**	0.25	0.22
CD8^+^CD28^−^CD57^+^	−0.18	0.17	−0.05	0.76	−0.27	0.17

Spearman correlation coefficients (r_s_) and *p* values for CMV^−^ and CMV^+^ persons and for the whole cohort are shown. p < 0.05 was considered significant. For all subpopulations, N_CMV_^−^ = 33, N_CMV_^+^ = 26, N_all_ = 59. Detailed gating strategy used to define the populations is shown in Fig. 3

Statistically significant values are shown in bold

### The phenotype of CD8^+^ T cell subsets change with BMI

**Fig. 4:**
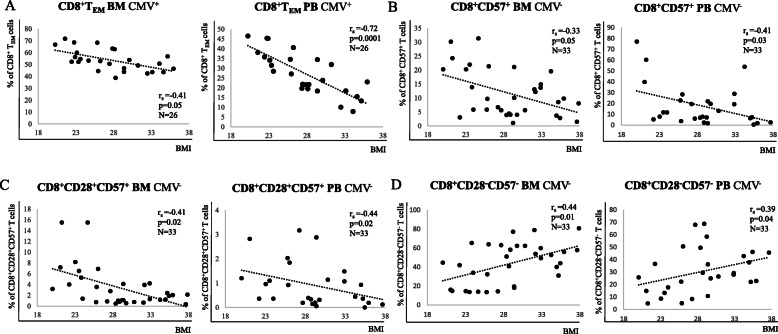
Summary of the significant correlations in paired BM/PB samples. Relationship between (**a**) CD8^+^ T_EM_ (CMV^+^), (**b**) CD8^+^CD57^+^ (CMV^−^), (**c**) CD8^+^CD28^+^CD57^+^ (CMV^−^) and (**d**) CD8^+^CD28^+^CD57^+^ (CMV^−^) T cells in BMMCs and PBMCs and BMI. Spearman coefficient (r_s_), sample number (N), and *p*-values are reported in each graph

To investigate whether, in parallel with their frequency, the phenotype of CD8^+^ T cell subsets may change with increased body weight, the expression of molecules involved in T cell activation/exhaustion, responsiveness to T cell cytokines and memory as well as senescence was measured in paired BMMC and PBMC samples using flow cytometry (Table 4 and Suppl. Table 4). Representative FACS plots are shown in Suppl. Fig. 4. In the whole CD8^+^ T cell population in the BM, PD-1 was reduced in overweight compared to lean persons only in the CMV^−^ group (Table 4). While no correlation was found between PD-1 expression within CD8^+^ T_CM_ and CD8^+^ T_EMRA_ cells and BMI, reduced levels of this molecule could be observed in the CD8^+^ T_EM_ subset with higher body weight in CMV^−^ persons. When the four CD28^+/−^CD57^+/−^ subsets were considered, the negative correlation between PD-1 expression and BMI was shown in CD28^+^CD57^−^ CD8^+^ T cells, but not in the other subpopulations. Similar results were obtained in the PB (Suppl. Table 4). Again, PD-1 levels within CD8^+^ T cell subsets were negatively associated to BMI. In the whole CD8^+^ T cell population in the BM, the expression of IL-7Rα negatively correlated with BMI in CMV^−^ persons (Table 4). In CMV^+^ persons, overall levels were lower in comparison to CMV^−^ individuals (data not shown). The same results could be found in paired PBMC samples (Suppl. Table 4). The combination of the markers IL-7Rα and KLRG-1 within CD8^+^ T cells allows the definition of IL-7Rα^+^KLRG^−^ 1^−^ memory progenitor effector cells (MPEC), which are known to differentiate into memory cells, and IL-7Rα^−^KLRG^−^ 1^+^ short living effector cells (SLEC), which may either die or accumulate as senescent-like T cells [21, 22]. In the BM of both CMV^−^ and CMV^+^ persons, MPEC were negatively associated with BMI, while the levels of SLEC did not change. In the PB, MPEC decreased in donors with higher BMI only in the CMV^+^ group (Suppl. Table 4). Interestingly, the expression of KLRG-1, commonly associated with terminally differentiated cells [23], positively correlated with BMI when its expression was measured within CD8^+^CD28^−^ and CD8^+^ T_EMRA_ cells in CMV^+^ persons. Similar results could be obtained for the PB (Suppl. Table 4). A summary about correlations regarding CD8^+^ T cell subsets which were significant in both BM and PB is shown in Fig. 4. Representative FACS plots summarizing the differences between lean and obese donors in paired BM and PB samples are shown in Suppl. Fig. 5.

**Table 4:** Correlations between expression of PD-1, IL-7Rα and KLRG-1 within CD8^+^ T cell subsets in the human BM with BMI

	all		CMV^**−**^		CMV^**+**^	
	r_s_	*p* value	rs	*p* value	rs	*p* value
**CD8**^**+**^**PD-1**^**+**^	−0.15	0.27	**−0.34**	**0.04**	−0.01	0.96
**CD8**^**+**^**IL-7Rα**^**+**^	−0.17	0.24	**−0.33**	**0.05**	−0.03	0.88
**CD8**^**+**^**MPEC**	**−0.30**	**0.04**	**−0.45**	**0.03**	**−0.37**	**0.04**
CD8^+^ SLEC	0.23	0.14	0.32	0.14	0.34	0.11
**CD8**^**+**^**CD28**^**+**^**KLRG-1**^**+**^	**0.32**	**0.04**	0.29	0.21	0.33	0.13
**CD8**^**+**^**CD28**^**−**^**KLRG-1**^**+**^	**0.33**	**0.03**	0.21	0.37	**0.46**	**0.03**
PD-1^+^ CD8^+^T_CM_	−0.10	0.43	−0.15	0.42	−0.06	0.78
**PD-1**^**+**^**CD8**^**+**^**T**_**EM**_	**−0.27**	**0.04**	**−0.44**	**0.01**	−0.10	0.62
KLRG1^+^CD8^+^T_EM_	0.17	0.25	0.09	0.69	0.16	0.48
**CD57**^**+**^**CD8**^**+**^**T**_**EM**_	**−0.28**	**0.03**	− 0.32	0.07	− 0.13	0.52
PD-1^+^CD8^+^T_EMRA_	−0.08	0.86	−0.15	0.68	−0.07	0.92
**KLRG1**^**+**^**CD8**^**+**^**T**_**EMRA**_	**0.37**	**0.01**	0.32	0.15	**0.55**	**0.01**
CD57^+^CD8^+^T_EMRA_	−0.29	0.45	**− 0.38**	**0.03**	− 0.28	0.19
**CD28**^**+**^**CD57**^**−**^**PD-1**^**+**^	**−0.31**	**0.02**	**−0.47**	**0.01**	−0.20	0.34
CD28^+^CD57^+^ PD-1^+^	−0.15	0.25	−0.24	0.17	0.09	0.66
CD28^−^CD57^−^ PD-1^+^	−0.07	0.56	−0.24	0.18	0.08	0.70
CD28^−^CD57^+^ PD-1^+^	−0.08	0.96	−0.18	0.31	0.16	0.42

Spearman correlation coefficients (r_s_) and *p* values for CMV^−^ and CMV^+^ persons and for the whole cohort are shown. p < 0.05 was considered significant. For all subpopulations, N_CMV_^−^ = 33, N_CMV_^+^ = 26, N_all_ = 59. Detailed gating strategy used to define the populations and representative FACS plots are shown in Fig. 3 and Supplementary Fig. 3

Statistically significant values are shown in bold

In summary, our results indicate that responsiveness to IL-7 and expression of PD-1 and KLRG-1 change with increased BMI, in both BM and PB. In addition, memory CD8^+^ T cells may be reduced in overweight persons.

## Discussion

Whether obesity may be considered a disease is still under debate [24]. Despite this, it is now evident that increased body weight is linked to a broad spectrum of diseases, which overall reduce healthspan in the elderly. Several studies showed that immune cell types such as macrophages, innate lymphoid cells, T cells and B cells are contained within the adipose tissue [25–28]. In particular, lymphoid cells such as NK cells, NKT, and B and T cells may play a fundamental role in the inflammatory process associated with obesity. Among the different types of adipose tissues present in the body, MAT has recently gained considerable importance in the field of immunological memory [18]. In our lab, we recently reported that MAT can produce survival factors for adaptive immune cells, therefore playing an important role in establishing niches for memory T cells and long-lived plasma cells in the BM. Despite this, as MAT additionally produces pro-inflammatory molecules such as IL-6 and TNF, its accumulation may be detrimental for the maintenance of immunological memory in the BM. Indeed, reduced plasma cell function with increased numbers of adipocytes has been documented [18].

The first aim of the current study was to assess whether the BM niche environment supporting the maintenance of adaptive immune cells may change with increased BMI (Fig. 1). Although the focus of our work was not on MAT but on BMMCs, a close interaction between BM adipocytes and other BM cells is expected. Increased production of IL-15, mainly important for the survival of highly differentiated T cells, and IL-6, a pro-inflammatory molecule additionally involved in plasma cell and T cell maintenance, was found in overweight compared to lean persons. In addition, a positive correlation was found between ROS in BMMCs and BMI, in line with the observations that obese persons are characterized by high levels of oxidative stress [3]. Increased oxygen radicals found in obese persons were linked to a decreased production of pro-inflammatory molecules by CD8^+^ T cells. Indeed, we saw a negative correlation between IFNγ^+^ and TNF^+^CD8^+^ T cells and BMI in the BM, suggesting that CD8^+^ T cell effector functions in the BM may be impaired in overweight persons. This situation could be observed in the BM but not in the PB. This aspect slightly differs from the situation observed in the BM during aging [14]. Although, also in this case, an increase in the levels of IL-15, IL-6 and ROS was documented, the expression of IFNγ and TNF was increased. We can speculate that, in the BM of obese persons, other factors not considered in the current study (such as adipokines or other molecules) may counteract the production of IFNγ and TNF.

Another important aspect to investigate is whether the frequency of immune cell subsets in the BM change with increased BMI. For this part, the influence of CMV also needs to be considered, and therefore the samples were divided into CMV^−^ and CMV^+^ groups. CMV is a persisting herpes virus, which, depending on the cohort, is present in 60–100% of individuals [29, 30]. By itself, CMV has been linked to diseases and/or pathologies such as cardiovascular diseases and cancer [31–33]. More recently, we described how CMV affects the phenotype of subsets of highly differentiated CD8^+^ T cells in the BM [15]. After a first general definition of immune cell populations in the BM, we observed that both CD4^+^ T cells and B cells significantly increased in persons with high BMI, but only in the CMV^−^ group. Higher frequency of both subsets in the PB of obese persons has been documented [8]. Activated CD4^+^ T cells accumulate in WAT of mice on high fat diet, supporting the recruitment of M1 macrophages with a pro-inflammatory phenotype, therefore leading to WAT inflammation [34]. Although it is still unknown whether CD4^+^ T cells may additionally infiltrate into MAT, we can speculate that this subset may contribute to the onset of a pro-inflammatory environment within the marrow. As B cell frequency was described to correlate with BMI, further studies must be performed in order to assess whether specific B cell subsets may change with increased body weight.

As a next step, we investigated whether the phenotype of “classical” CD8^+^ and CD4^+^ T cell subsets in the BM may be influenced by BMI. This and the following parts were performed in both BM and PB in paired samples. Interestingly, most of the significant correlations within CD8^+^ T cell subpopulations in the BM were found in CMV^−^ persons. This suggests that the CMV^+^ group may be more homogeneous regarding CD8^+^ T cell parameters, and the effects of obesity may be more evident in CMV^−^ persons. In alternative, in CMV^+^ donors, the impact of CMV on the T cell compartment may be so profound that other more subtle changes such as BMI may be missed. CD8^+^ T_CM_ and CD8^+^ T_EM_ subsets are known to include bona fide memory cells, which are classically characterized by expression of costimulatory receptor CD28 and lack the marker of terminal differentiation CD57. Overall, our results indicate that BM CD8^+^ T_CM_ and CD8^+^ T_EM_ decreased in overweight compared to lean persons, in either the CMV^−^ (CD8^+^ T_CM_) or in the CMV^+^ (CD8^+^ T_EM_) groups. In addition, the frequency of IL7Rα^+^KLRG-1^−^ MPEC, known to differentiate into memory T cells [21, 22], was negatively associated with BMI in the BM of both CMV^−^ and CMV^+^ persons. Similar trends were described for the PB, although no differences were found in the CMV^−^ group. Altogether, these data indicate that the maintenance of memory CD8^+^ T cells in the BM may be negatively affected by body weight. In addition, it is still unknown whether MAT may be involved in the competition for space between immune cell populations in the BM, supporting the preferential accumulation of certain subsets [35, 36].

We recently reported that, using the markers CD28 and CD57, populations of non-activated/early-activated/memory CD28^+^CD57^−^, activated/immune regulatory-like CD28^+^CD57^+^, activated/early-senescent CD28^−^CD57^−^ and terminally differentiated/senescent-like CD28^−^CD57^+^ cells could be identified within CD8^+^ T cells [37]. Although the overall amount of CD8^+^CD57^+^ T cells decreased while the frequency of CD28^−^CD8^+^ T cells did not change in relationship to BMI, differences were observed when the four subsets were considered. Interestingly, both the CD28^+^CD57^−^ population, which includes memory CD8^+^ T cells, and the CD28^+^CD57^+^ subset, which may play an important role in regulating immune responses [37], decreased in the BM of overweight compared to lean persons in the CMV^−^ group. Despite this, although a link between obesity and T cell senescence has been suggested in murine adipose tissue [38], only few senescent-like populations were influenced by body weight in our cohort, in both BM and PB. In particular, while CD28^−^CD57^+^, IL7Rα^−^KLRG-1^+^ SLEC and CD8^+^T_EMRA_ T cells did not change, only KLRG-1 expression positively correlated with BMI in some subsets. Despite this, it is important to consider that the expression of KLRG-1 alone may not represent an optimal marker for T cell senescence [39]. We can clearly observe that the frequency of CD28^−^CD57^−^ CD8^+^ T cells, which accumulates in the BM compared to PB, significantly increased with body weight. Thus, we can speculate that, although the “early steps” of CD8^+^ T cell senescence may be triggered by obesity, the “late steps”, which involve the upregulation of CD57, may be somehow inhibited. The reduced expression of IFNγ and TNF by CD8^+^ T cells may partially be explained by the expansion of CD28^−^CD57^−^ CD8^+^ T cells in obese individuals, as low production of both cytokines was described in this subset [37].

Furthermore, in CMV^−^ persons, PD-1 expression on BM CD8^+^ T cell subsets is negatively associated to BMI. As this co-inhibitory molecule is expressed by activated T cells and inhibits their further activation (therefore making T cells “exhausted”) [40], CD8^+^ T cells may be less activated in obese persons.

We next investigated whether some relationships could be found between phenotype of CD4^+^ T cell subsets and BMI, in the BM and in the PB. In this case, significant differences were obtained only in the BM, indicating that the BM environment may specifically influence CD4^+^ T cell parameters. Interestingly, only in CMV^+^ persons, CD4^+^ T_N_ and CD4^+^ T_CM_ increased while CD4^+^ T_EM_ decreased in overweight persons. While T_N_ and T_CM_ are stable, T_EM_ cells are known to display rapid turnover [41]. Thus, our data suggest that, in obese CMV^+^ individuals, the maintenance of naïve and long-lived memory CD4^+^ T cells in the BM may be improved. Whether this aspect may be linked with the expansion of MAT observed with increased body weight must be investigated in future studies. As observed for CD8^+^ T cells, the effector functions of BM CD4^+^ T cells may be impaired with obesity, as reduced expression of activation/exhaustion marker PD-1 could be observed. Although the levels of BM CD4^+^ T_EMRA_ positively correlated with BMI in the whole cohort, other senescent-like CD4^+^ T cell subsets were not associated with body weight. Indeed, no correlations were observed when the markers CD28 and CD57 were considered.

## Conclusion

Our study describes for the first time that the maintenance of memory T cell subsets in the BM may change in relationship to BMI. Thus, in addition to aging and CMV, BMI represents a further parameter to consider, particularly when the phenotype of effector/memory T cells is studied. Metabolic interventions must be planned, in order to assess whether the situation described in overweight persons may be reversible, therefore improving the fitness of adaptive immune cells.

## Supplementary information

**Additional file 1.**

**Additional file 2.**

## Data Availability

All datasets and material are available upon request.

## Abbreviations

BMI: body mass index
PB: peripheral blood
PBMC: PB mononuclear cells
BM: bone marrow
BMMCs: BM mononuclear cells
ROS: reactive oxygen species
