# Supplementary material for: The impact of body mass index on adaptive immune cells in the human bone marrow
Source: Immun Ageing. 2020 May 27;17:15. doi: 10.1186/s12979-020-00186-w (PMC7251898; doi:10.1186/s12979-020-00186-w)
Supplement: Supplementary file 1 — Additional file 1. [file 12979_2020_186_MOESM1_ESM.docx]

| **Surface antibody** | **Clone** | **Fluorochrome** | **Company** |
| --- | --- | --- | --- |
| CD45 | HI30 | BV500 | BD Biosciences |
| CD16 | 3G8 | FITC | BD Biosciences |
| CD56 | NCAM16.2 | PE | BD Biosciences |
| CD14 | HCD14 | Pe-Cy7 | Biolegend |
| CD19 | HIB19 | PE | BD Biosciences |
| CD138 | 44F9 | APC | Miltenyi |
| CD38 | HIT2 | BV500 | BD Biosciences |
| CD3 | REA613 | VioGreen | Miltenyi |
| CD3 | REA613 | APC-Vio770 | Miltenyi |
| CD4 | REA623 | VioGreen | Miltenyi |
| CD8 | REA734 | PE-Vio770 | Miltenyi |
| CD28 | CD28.2 | BV421 | BD Biosciences |
| CD57 | TB03 | APC | Miltenyi |
| CCR7 | REA546 | FITC | Miltenyi |
| CD45RA | HI100 | PerCp | BD Biosciences |
| PD-1 | EH12.2H7 | PE | Biolegend |
| IL-7Rα | eBioRDR5 | APC | eBioscience |
| KLRG-1 | 2F1/KLRG1 | PE | Biolegend |
| **Intracellular antibody** | **Clone** | **Fluorochrome** | **Company** |
| IFNγ | B27 | APC | BD Biosciences |
| TNF | Mab11 | FITC | BD Biosciences |
| IL-15 | 34559 | APC | R&D Systems |
| IL-6 | MQ2-13A5 | APC | eBioscience |

**Suppl.Table 1**. Antibody used for the flow cytometry stainings.

|  | **all** | | **CMV^-^** | | **CMV^+^** | |
| --- | --- | --- | --- | --- | --- | --- |
|  | r_s_ | p value | r_s_ | p value | r_s_ | p value |
| CD4^+^ T cells (of CD3^+^) | 0.20 | 0.26 | 0.38 | 0.09 | 0.10 | 0.69 |
| CCR7^+^ CD45RA^+^ (CD4^+^T_N_) | 0.053 | 0.75 | 0.09 | 0.69 | 0.08 | 0.75 |
| CCR7^+^ CD45RA^-^ (CD4^+^T_CM_) | -0.17 | 0.31 | 0.07 | 0.77 | -0.31 | 0.20 |
| CCR7^-^CD45RA^-^ (CD4^+^T_EM_) | -0.04 | 0.79 | 0.16 | 0.51 | 0.04 | 0.88 |
| CCR7^-^CD45RA^+^ (CD4^+^_TEMRA_) | 0.32 | 0.05 | 0.22 | 0.34 | 0.39 | 0.09 |
| CD4^+^CD28^-^ | 0.13 | 0.43 | 0.00 | 0.99 | 0.21 | 0.38 |
| CD4^+^CD57^+^ | 0.27 | 0.10 | 0.11 | 0.64 | 0.34 | 0.15 |
| CD4^+^PD-1^+^ | -0.19 | 0.24 | 0.32 | 0.16 | -0.08 | 0.75 |
| CD4^+^IL-7Rα^+^ | 0.12 | 0.48 | 0.09 | 0.70 | -0.01 | 0.75 |
| PD-1^+^ CD4^+^T_CM_ | 0.17 | 0.31 | 0.06 | 0.79 | 0.14 | 0.58 |
| PD-1^+^CD4^+^T_EM_ | -0.13 | 0.42 | 0.27 | 0.25 | -0.09 | 0.72 |
| CD57^+^CD4^+^T_EM_ | 0.20 | 0.21 | 0.06 | 0.79 | 0.20 | 0.41 |
| PD-1^+^CD4^+^T_EMRA_ | -0.06 | 0.74 | 0.16 | 0.51 | -0.01 | 0.97 |
| CD57^+^CD4^+^T_EMRA_ | 0.19 | 0.23 | 0.02 | 0.92 | 0.35 | 0.15 |

**Suppl.Table 2**. Correlations between CD4^+^ T cell subsets, expression of PD-1 and IL-7Rα within CD4^+^ T cell subsets in the PB and BMI. Spearman correlation coefficients (r_s_) and p values for CMV^-^ and CMV^+^ persons and for the whole cohort are shown. p<0.05 was considered significant. For all subpopulations, N_CMV_^-^ =20, N_CMV_^+^ =19, N_all_=39.

|  | **all** | | **CMV^-^** | | **CMV^+^** | |
| --- | --- | --- | --- | --- | --- | --- |
|  | r_s_ | p value | rs | p value | rs | p value |
| CD8^+^ T cells | -0.15 | 0.26 | -0.12 | 0.55 | -0.14 | 0.50 |
| **CCR7^+^ CD45RA^+^ (CD8^+^T_N_)** | **0.34** | **0.02** | 0.24 | 0.29 | 0.35 | 0.10 |
| CCR7^+^ CD45RA^-^ (CD8^+^T_CM_) | -0.07 | 0.66 | -0.05 | 0.84 | -0.17 | 0.44 |
| **CCR7^-^CD45RA^-^ (CD8^+^T_EM_)** | **-0.41** | **0.005** | 0.001 | 0.99 | **-0.72** | **0.0001** |
| CCR7^-^CD45RA^+^ (CD8^+^_TEMRA_) | -0.22 | 0.15 | -0.31 | 0.16 | -0.11 | 0.58 |
| **CD8^+^CD57^+^** | **-0.34** | **0.01** | **-0.41** | **0.03** | -0.29 | 0.15 |
| CD8^+^CD28^-^ | -0.12 | 0.45 | -0.23 | 0.31 | 0.15 | 0.50 |
| CD8^+^CD28^+^CD57^-^ | 0.01 | 0.96 | -0.04 | 0.85 | -0.06 | 0.77 |
| **CD8^+^CD28^+^CD57^+^** | -0.18 | 0.18 | **-0.44** | **0.02** | -0.03 | 0.86 |
| **CD8^+^CD28^-^CD57^-^** | **0.27** | **0.05** | **0.39** | **0.04** | 0.25 | 0.22 |
| CD8^+^CD28^-^CD57^+^ | -0.25 | 0.06 | -0.30 | 0.12 | -0.24 | 0.23 |

**Suppl. Table 3**. Correlations of CD8^+^ T cell subsets in in the human PB with BMI. Spearman correlation coefficients (r_s_) and p values for CMV^-^ and CMV^+^ persons and for the whole cohort are shown. p<0.05 was considered significant. For all subpopulations, N_CMV_^-^ =28, N_CMV_^+^ =26, N_all_=55.

|  | **all** | | **CMV^-^** | | **CMV^+^** | |
| --- | --- | --- | --- | --- | --- | --- |
|  | r_s_ | p value | rs | p value | rs | p value |
| **CD8^+^PD-1^+^** | **-0.38** | **0.005** | **-0.57** | **0.002** | -0.31 | 0.13 |
| **CD8^+^IL-7Rα^+^** | -0.13 | 0.33 | **-0.39** | **0.04** | 0.01 | 0.96 |
| **CD8^+^ MPEC** | -0.20 | 0.20 | 0.02 | 0.96 | **-0.39** | **0.05** |
| CD8^+^ SLEC | 0.07 | 0.62 | 0.09 | 0.70 | 0.29 | 0.18 |
| **CD8^+^CD28^+^KLRG-1^+^** | **0.43** | **0.003** | **0.47** | **0.03** | 0.37 | 0.08 |
| **CD8^+^CD28^-^KLRG-1^+^** | **0.49** | **0.001** | 0.04 | 0.87 | **0.61** | **0.002** |
| **PD-1^+^ CD8^+^T_CM_** | **-0.37** | **0.01** | **-0.36** | **0.05** | **-0.46** | **0.02** |
| **PD-1^+^CD8^+^T_EM_** | **-0.37** | **0.01** | **-0.53** | **0.0003** | -0.32 | 0.11 |
| KLRG1^+^CD8^+^T_EM_ | 0.28 | 0.07 | 0.18 | 0.42 | 0.32 | 0.14 |
| **CD57^+^CD8^+^T_EM_** | -0.24 | 0.08 | **-0.41** | **0.03** | -0.08 | 0.67 |
| **PD-1^+^CD8^+^T_EMRA_** | **-0.27** | **0.05** | **-0.62** | **0.001** | -0.09 | 0.65 |
| KLRG1^+^CD8^+^T_EMRA_ | 0.28 | 0.09 | 0.36 | 0.09 | 0.35 | 0.10 |
| **CD57^+^CD8^+^T_EMRA_** | **-0.26** | **0.05** | **-0.37** | **0.05** | -0.27 | 0.41 |
| **CD28^+^CD57^-^ PD-1^+^** | **-0.44** | **0.001** | **-0.64** | **0.0002** | -0.34 | 0.09 |
| CD28^+^CD57^+^ PD-1^+^ | -0.08 | 0.54 | -0.10 | 0.60 | -0.13 | 0.53 |
| **CD28^-^CD57^-^ PD-1^+^** | **-0.41** | **0.002** | **-0.68** | **< 0.0001** | -0.28 | 0.16 |
| **CD28^-^CD57^+^ PD-1^+^** | -0.15 | 0.29 | **-0.52** | **< 0.0001** | -0.02 | 0.91 |

**Suppl. Table 4**. Correlations between expression of PD-1, IL-7Rα and KLRG-1 within CD8^+^ T cell subsets in the human PB with BMI. Spearman correlation coefficients (r_s_) and p values for CMV^-^ and CMV^+^ persons and for the whole cohort are shown. p<0.05 was considered significant. For all subpopulations, N_CMV_^-^ =28, N_CMV_^+^ =26, N_all_=54.
