# Supplementary material for: The impact of body mass index on adaptive immune cells in the human bone marrow
Source: Immun Ageing. 2020 May 27;17:15. doi: 10.1186/s12979-020-00186-w (PMC7251898; doi:10.1186/s12979-020-00186-w)
Supplement: Supplementary file 2 — Additional file 2. [file 12979_2020_186_MOESM2_ESM.pptx]

## Slide 1
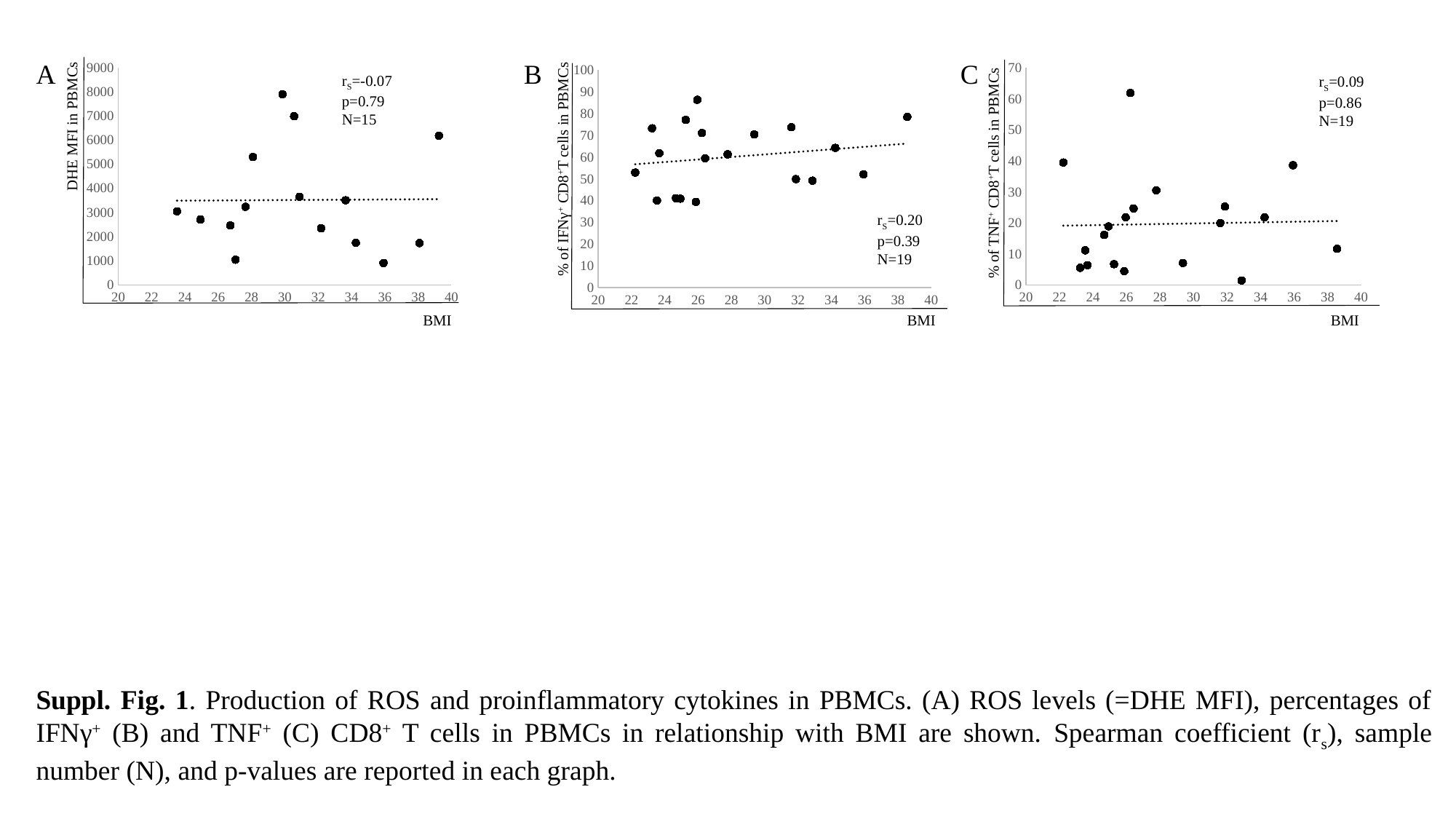

### Chart
| Category | PBMC ROS |
|---|---|rS=-0.07
p=0.79
N=15
DHE MFI in PBMCs
BMI
A
B
C
### Chart
| Category | TNF |
|---|---|
### Chart
| Category | IFN |
|---|---|rS=0.09
p=0.86
N=19
% of IFNγ+ CD8+T cells in PBMCs
% of TNF+ CD8+T cells in PBMCs
rS=0.20
p=0.39
N=19
BMI
BMI
Suppl. Fig. 1. Production of ROS and proinflammatory cytokines in PBMCs. (A) ROS levels (=DHE MFI), percentages of IFNγ+ (B) and TNF+ (C) CD8+ T cells in PBMCs in relationship with BMI are shown. Spearman coefficient (rs), sample number (N), and p-values are reported in each graph.

## Slide 2
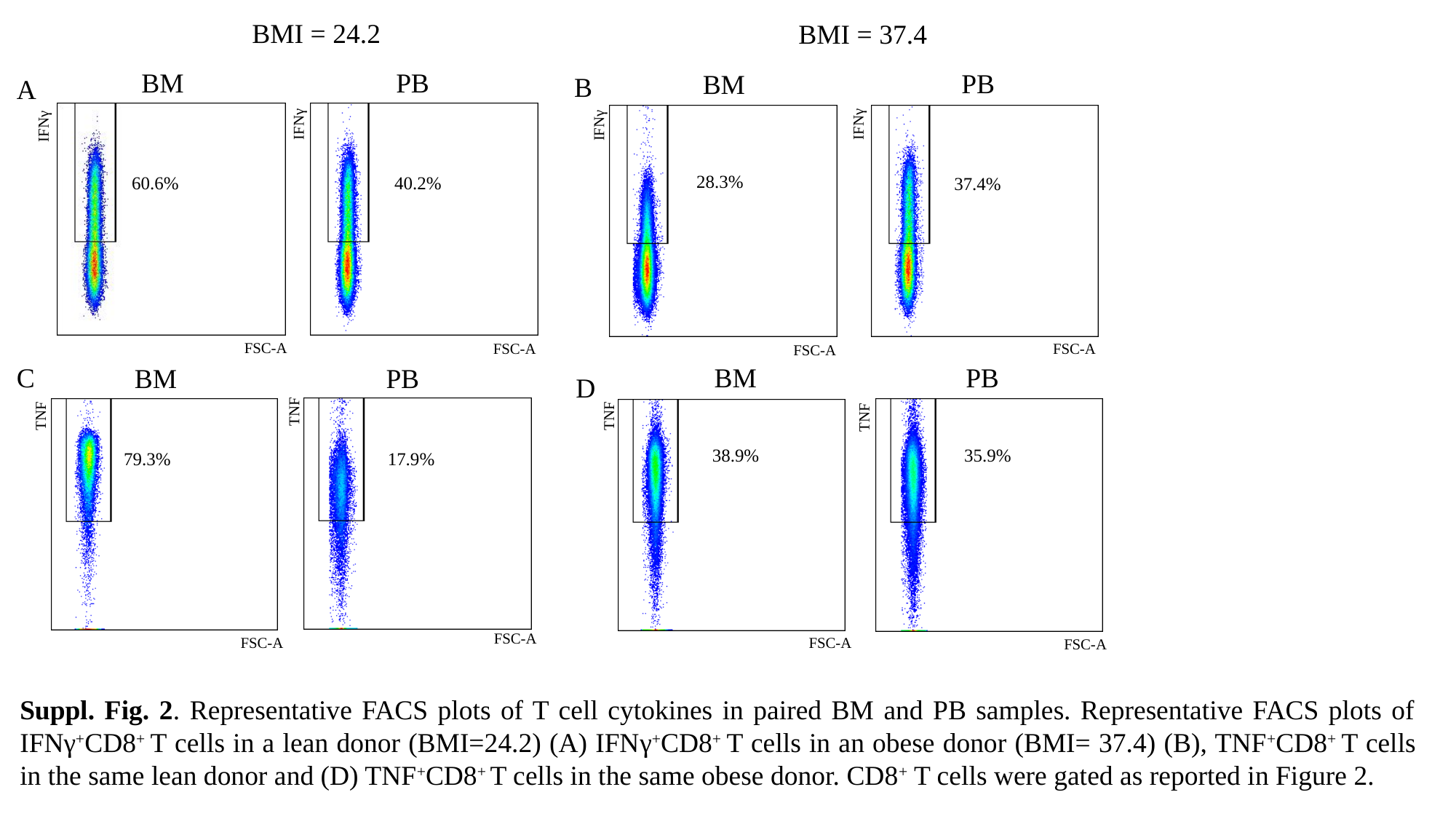

BMI = 24.2
BMI = 37.4
IFNγ
37.4%
FSC-A
BM
IFNγ
28.3%
FSC-A
PB
PB
IFNγ
40.2%
FSC-A
BM
IFNγ
60.6%
FSC-A
B
A
TNF
17.9%
FSC-A
TNF
79.3%
FSC-A
BM
PB
BM
PB
TNF
TNF
38.9%
35.9%
FSC-A
FSC-A
C
D
Suppl. Fig. 2. Representative FACS plots of T cell cytokines in paired BM and PB samples. Representative FACS plots of IFNγ+CD8+ T cells in a lean donor (BMI=24.2) (A) IFNγ+CD8+ T cells in an obese donor (BMI= 37.4) (B), TNF+CD8+ T cells in the same lean donor and (D) TNF+CD8+ T cells in the same obese donor. CD8+ T cells were gated as reported in Figure 2.

## Slide 3
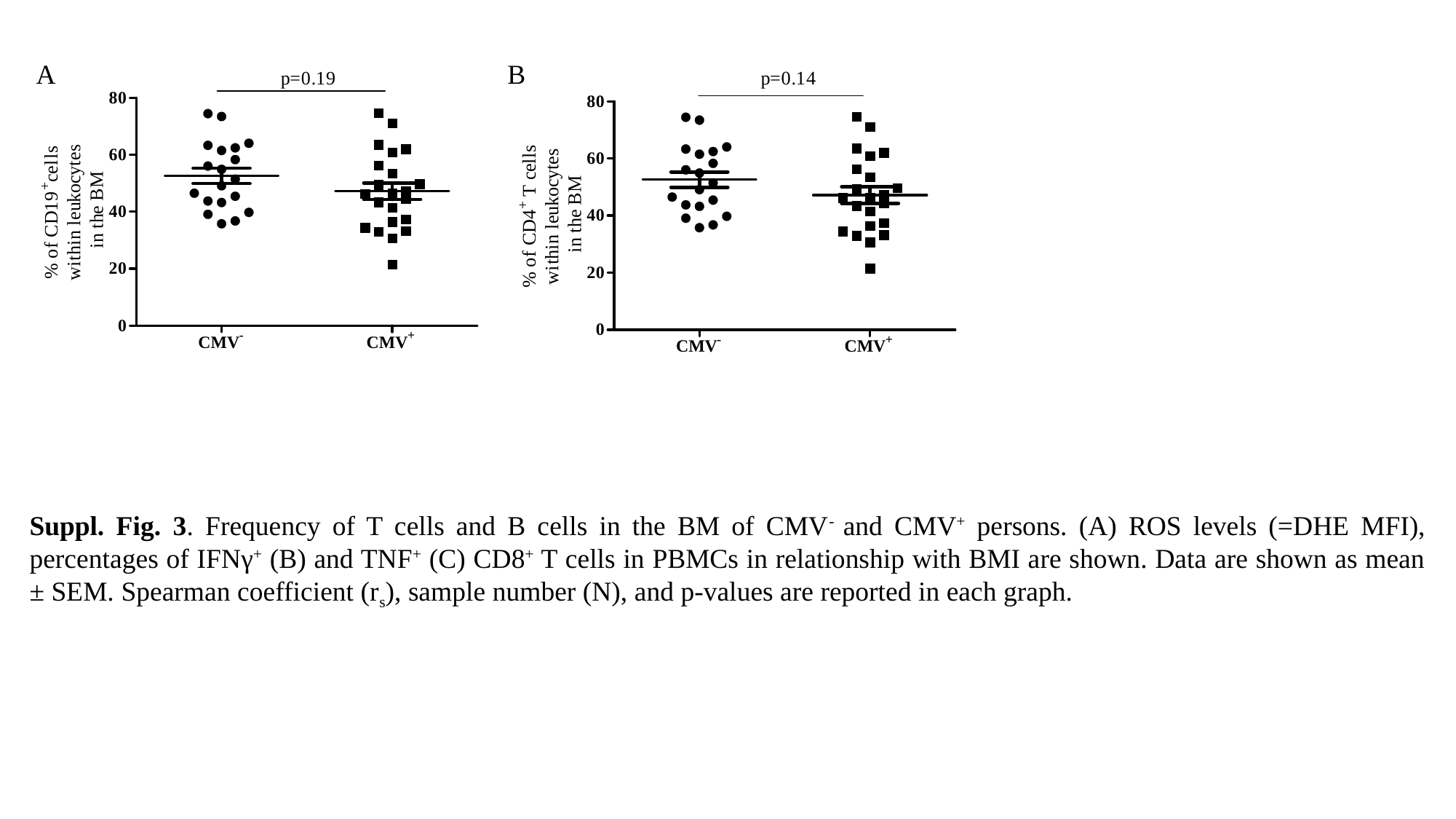

A
B
Suppl. Fig. 3. Frequency of T cells and B cells in the BM of CMV- and CMV+ persons. (A) ROS levels (=DHE MFI), percentages of IFNγ+ (B) and TNF+ (C) CD8+ T cells in PBMCs in relationship with BMI are shown. Data are shown as mean ± SEM. Spearman coefficient (rs), sample number (N), and p-values are reported in each graph.

## Slide 4
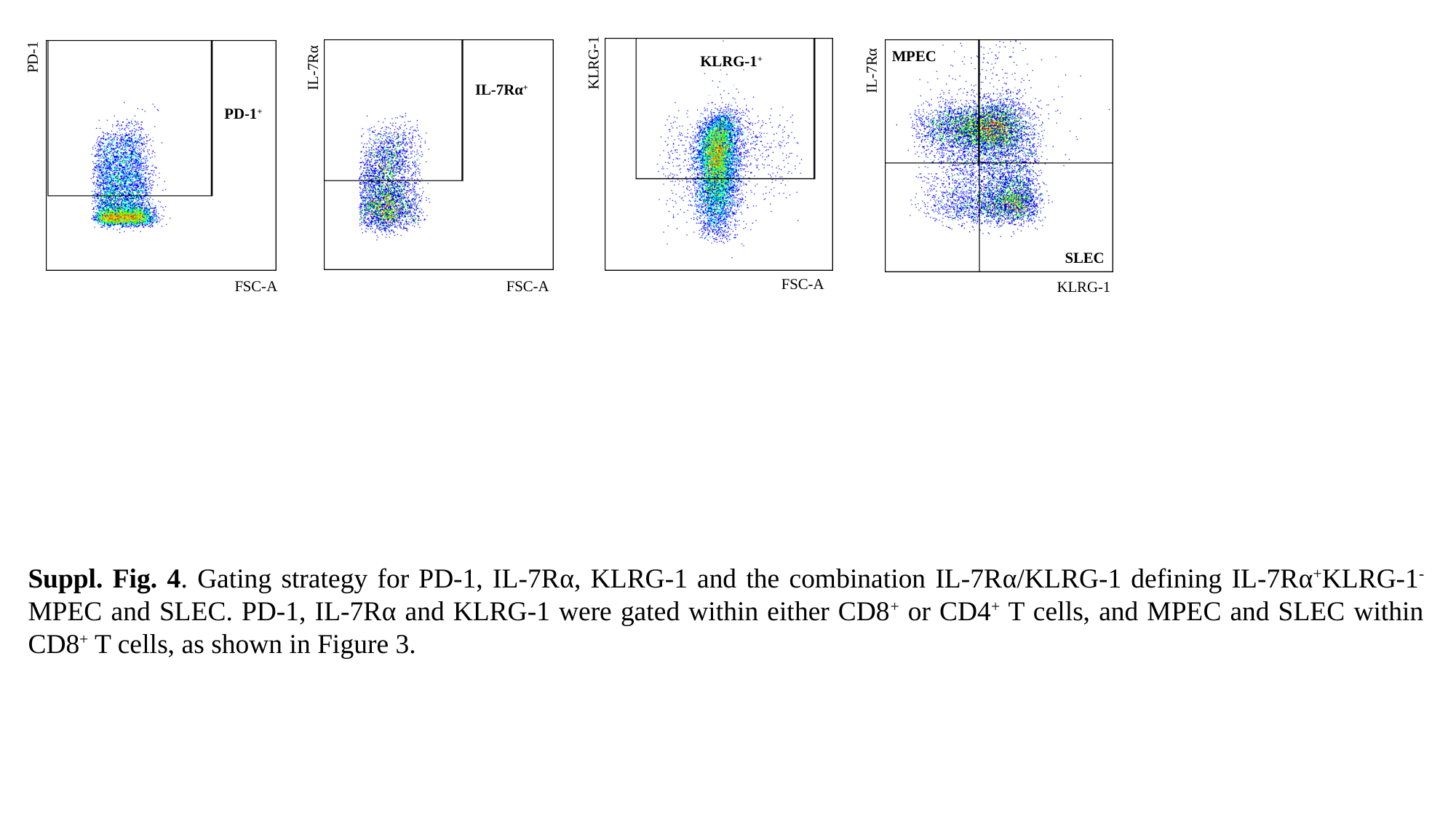

PD-1
PD-1+
FSC-A
KLRG-1
FSC-A
KLRG-1+
IL-7Rα
IL-7Rα+
FSC-A
IL-7Rα
MPEC
SLEC
KLRG-1
Suppl. Fig. 4. Gating strategy for PD-1, IL-7Rα, KLRG-1 and the combination IL-7Rα/KLRG-1 defining IL-7Rα+KLRG-1- MPEC and SLEC. PD-1, IL-7Rα and KLRG-1 were gated within either CD8+ or CD4+ T cells, and MPEC and SLEC within CD8+ T cells, as shown in Figure 3.

## Slide 5
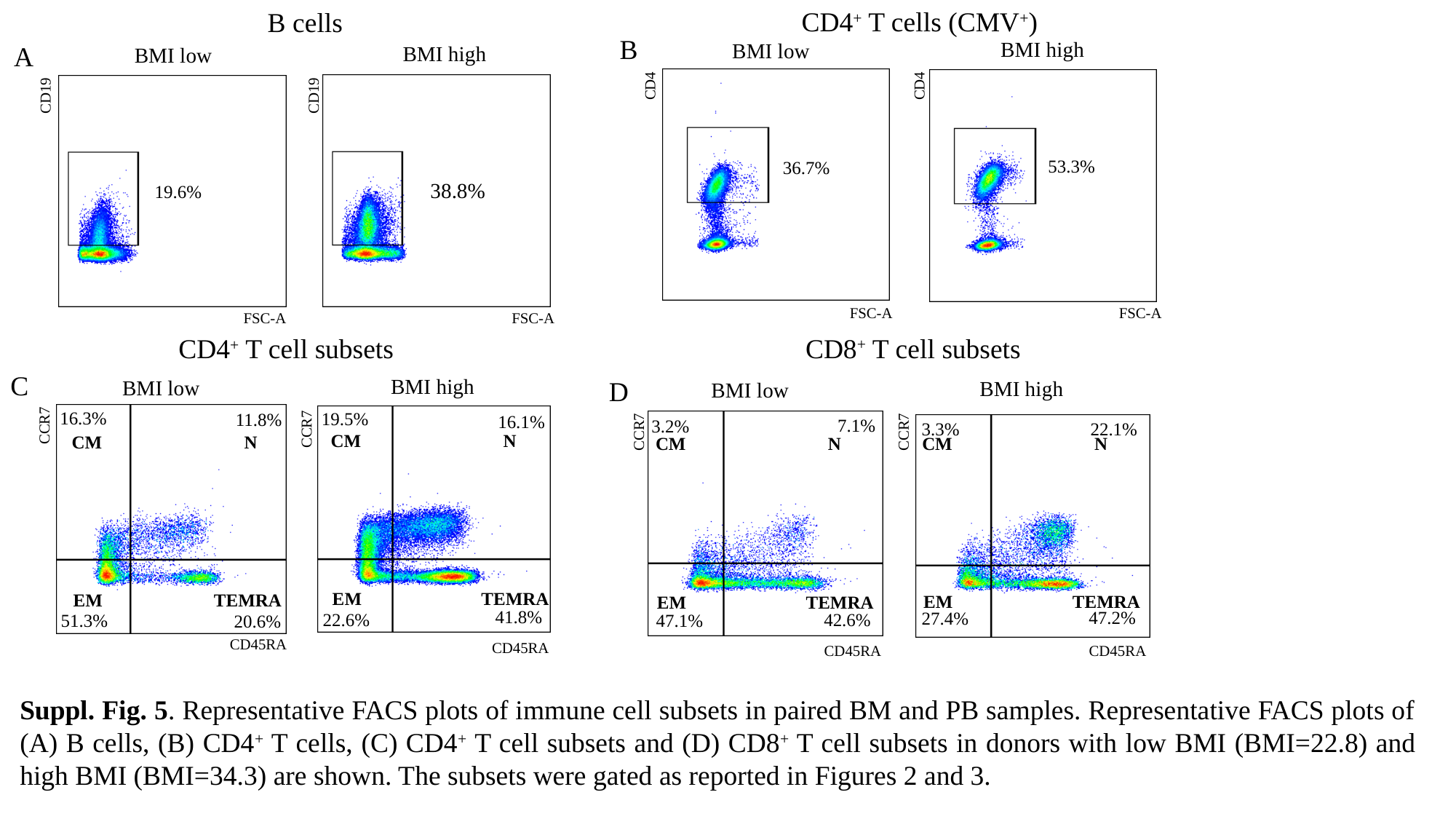

CD19
19.6%
FSC-A
CD19
38.8%
FSC-A
A
B cells
CD4
CD4
53.3%
36.7%
FSC-A
FSC-A
B
BMI high
BMI low
CD4+ T cells (CMV+)
BMI high
BMI low
CD8+ T cell subsets
CCR7
3.3%
22.1%
47.2%
27.4%
CM
N
TEMRA
EM
CD45RA
7.1%
3.2%
42.6%
47.1%
CM
N
TEMRA
EM
CCR7
CD45RA
BMI high
BMI low
D
CD4+ T cell subsets
CCR7
CCR7
16.3%
11.8%
51.3%
20.6%
CM
N
TEMRA
EM
19.5%
16.1%
41.8%
22.6%
CM
N
TEMRA
EM
CD45RA
CD45RA
BMI high
BMI low
C
Suppl. Fig. 5. Representative FACS plots of immune cell subsets in paired BM and PB samples. Representative FACS plots of (A) B cells, (B) CD4+ T cells, (C) CD4+ T cell subsets and (D) CD8+ T cell subsets in donors with low BMI (BMI=22.8) and high BMI (BMI=34.3) are shown. The subsets were gated as reported in Figures 2 and 3.
